# Self-Reported Losses Versus Actual Losses in Online Gambling: An Empirical Study

**DOI:** 10.1007/s10899-016-9648-0

**Published:** 2016-11-04

**Authors:** Michael Auer, Mark D. Griffiths

**Affiliations:** 1 Office 404 Albany House, Neccton Ltd, 324 Regent Street, London, W1B 3HH UK; 2 International Gaming Research Unit, Psychology Department, Nottingham Trent University, Burton Street, Nottingham, NG1 4BU UK

**Keywords:** Responsible gambling, Behavioral tracking, Gambling expenditure, Pre-commitment

## Abstract

Many research findings in the gambling studies field rely on self-report data. A very small body of empirical research also suggests that when using self-report, players report their gambling losses inaccurately. The aim of the present study was to evaluate the differences between objective and subjective gambling spent data by comparing gambler’s actual behavioral tracking data with their self-report data over a 1-month period. A total of 17,742 Norwegian online gamblers were asked to participate in an online survey. Of those surveyed, 1335 gamblers answered questions relating to gambling expenditure that could be compared with their actual gambling behavior. The study found that the estimated loss self-reported by gamblers was correlated with the actual objective loss and that players with higher losses tended to have more difficulty estimating their gambling expenditure (i.e., players who spent more money gambling also appeared to have more trouble estimating their expenses accurately). Overall, the findings demonstrate that caution is warranted when using self-report data relating to amount of money spent gambling in any studies that are totally reliant on self-report data.

## Introduction

Many research findings in the gambling studies field—including research into online gambling—rely on self-report data. In a systematic review of online gambling studies, Shaffer et al. (2010) reported that out of 30 papers investigating internet gambling (excluding their own studies), none of them included studies concerning actual online gambling behavior. In recent years, a number of research teams in the gambling studies field have been given access to behavioral tracking data provided by online gambling operators and which have formed the basis of various published studies (e.g., Broda et al. 2008; Focal Research Consultants 2007; Afifi et al. 2014; Auer and Griffiths 2013, 2014, 2015). However, most studies still rely on data collected via self-recollected information (e.g., Wardle et al. 2011; Elton-Marshall et al. 2016) or experiments conducted in laboratory settings (e.g., Montes and Weatherly 2016).

The comparison between industry-reported data and player-reported data led Volberg et al. (2001) to conclude that players report their gambling losses inaccurately. The basis for their conclusion was the comparison of average self-reported past year losses with industry wide data of the same time period. Blaszczynski et al. (1997) studied the interpretation of seemingly straightforward survey items such as “how much do you spend gambling?” They found that only two-thirds or less of their participants calculated the figure to be the difference between the initial amount risked and the residual amount of money at the conclusion of the session. This led to the conclusion that more attention should be given to providing clear instructions in surveys. Wood and Williams (2007) asked about past month gambling expenditure in 12 different ways to a random sample of 2424 Ontario adult gamblers. The relative validity of each question format was subsequently established by the correspondence of reported gambling expenditures with actual Ontario gambling revenue, as well as with amounts obtained by prospective diaries. They found that slight variations in question wording resulted in significant variation in reported expenditure amounts.

Using any kind of aggregated data (e.g., self-reported information or industry data), it is impossible to investigate systematic biases about player spending. For instance, some players might overestimate their losses whereas others might underestimate their losses. Furthermore, such estimation biases may be associated with specific demographic variables such as age, gender, and/or the level of education. Various researchers (e.g., Blaszczynski et al. 2008; Williams and Wood 2004) have compared self-reported information of gambling expenditure with objective information. However, the comparisons were not made on an individual-player level (i.e., they reported pool spending outcomes). For instance, Williams and Wood (2004) received a complete month of self-recollected gambling expenditure from 344 study participants in Ontario, Canada. From this information, they projected the yearly spending of all Ontario residents. They compared this number with the objectively reported expenditure by the Ontario Lottery and Gaming Corporation (OLGC) and found reasonable support for the contention that subjective information is an accurate reflection of true expenditures. However, each single participant’s actual gambling expenditure was unknown.

To date, there has been only one study that has collected and compared self-reported gambling spend data with actual spend data (i.e., Braverman et al. 2014). Braverman et al. (2014) compared the accuracy of self-reported losses with actual loss information from a sample of 2259 *bwin* online players. They compared the accuracy of self-reported losses over a 3-month period and a 12-month period and found that players were more accurate when estimating their short-term losses. The estimation bias was also larger for players with gambling-related problems. However, no consistent pattern regarding overestimation and underestimation was determined.

Braverman et al. (2014) also studied the effect of gambling experience. The number of days within the time period when gamblers placed at least one bet was used as a proxy for gambling experience. In their view, the more often gamblers placed bets, the more experienced they were. The results showed that more experienced sports bettors and casino players reported more inaccurate estimates of gambling outcomes than less experienced gamblers. In order to determine whether the gamblers utilized fixed odds, live odds, or casino games, Braverman et al. (2014) relied upon player self-report. However, in reality, gamblers often engage in many different types of gambling games with varying intensity (Auer and Griffiths 2013). Despite these limitations, this study is the only one to date that has directly compared self-report spending data with objective (i.e., actual) data on an individual player level.

In specific circumstances, gambling behavior can be objectively measured, most notably in online gambling or in land-based gambling when gamblers use player cards and/or loyalty cards. In fact, gambling is one of the few consumptive (and potentially addictive) behaviors that can be objectively tracked. Such behavioral tracking is not possible in the areas of cigarette smoking or alcohol consumption and, therefore, has to rely on self-report data. However, there are areas where both self-report information and objective data have been subject to research (e.g., research examining physical activity). This is due to new technologies (e.g., smart watches) that can track bodily functions such as pulse rate and/or the number of steps walked or ran. Findings from areas outside of the gambling studies field (i.e., physical activity, obesity, sexual behavior) have shown that there is a mismatch between objective and subjective data when they are collected from the same person (Prince et al. 2008). The one study published in the gambling studies field (i.e., Braverman et al. 2014) only compared the objective and subjective data at an aggregated (rather than an individual) level. The present study collected subjective self-report gambling data (i.e., money individuals claimed they had spent gambling) and compared it to objective data (i.e., actual money spent gambling) from the same individual. The relationship between objective and subjective data was also examined across different demographic and behavioral characteristics. Consequently, the aim of the present study was to evaluate the differences between objective and subjective gambling expenditure in the same individuals. Furthermore, it was hypothesized that players who engaged in high event frequency games (e.g., casino-type games) would be less accurate in estimating their losses compared to those players who engaged in low event frequency games (e.g., lottery games) because the latter usually spend smaller amounts gambling.

## Method

### Participants and Procedure

At the beginning of June 2015, a sample of 17,742 online gamblers from the Norwegian online gambling site of the state-owned gambling operator *Norsk Tipping* were asked to participate in an online survey. Active players in the present study had to have played at least one online casino game in May 2015 in order to be included in the subsequent data analysis. Additionally, high spending gamblers were oversampled (therefore the 17,742 gamblers were not representative of the whole population of *Norsk Tipping* online gamblers). This is because the research team wanted to examine the differences between high and low intensity gamblers. A representative sample would have contained relatively few high spending gamblers. The survey was administered online via the *Norsk Tipping* gambling site and players were notified about the study in their online gambling account message inbox. In the present paper, only responses relating to gambling expenditure and awareness are reported. For instance, one survey item asked gamblers to estimate their overall loss or win during a 1-month period immediately prior to the survey. This was an open question in which participants could enter any amount of money. Out of the 17,742 gamblers who were surveyed, 1335 of them answered this question (7.5 %). As all gamblers playing *Norsk Tipping* games require a player card to participate, all games played and amount of money spent (both online and offline) can be assigned to a specific individual player. This means that each player’s gambling expenditure online as well as land-based gambling was known for the 1335 players that provided a subjective estimate for how much they won or lost gambling.

### Data Analysis

Given that the primary aim of the present study was to evaluate the differences between objective and subjective gambling expenditure, correlations between self-report and objective information were calculated. Given that correlations only assess the strength of a relation between two variables, not the agreement between them, Bland–Altman plots were also used to more accurately analyze the agreement between subjective and objective gambling expenditure. Calculations of the loss estimation bias between subjective and objective information were also made in relation to player-specific attributes such as gender, age, and gambling-type. Due to the skewed and non-normal distribution of the variables, non-parametric statistics such as the median and the quantiles were used to analyze gambling expenditure.

## Results

Table 1 shows the distribution of both the total sample of 17,742 players and the 1335 players that answered the survey question relating to how much money they had won or lost during May 2015. Table 1 shows that compared to the total sample, players who reported their subjective gambling spend were significantly (1) older (45 vs. 41 years), (2) less likely to be female (27 vs. 31 %), and (3) more likely to have been registered with *Norsk Tipping* longer (107 vs. 94 months). The median gross gaming revenue (GGR) which is the difference between the amount of money bet minus the amount of money won was larger among those that reported their subjective gambling spend compared to the total sample (1027 [Norwegian Krone] NOK. vs. 702 NOK). This means that 50 % of the players who reported their subjective gambling spend said they lost more than 1027 NOK in May 2015 and 50 % lost less. The same pattern was observed for the amount of money bet (1941 NOK vs. 1220 NOK). The distribution of the variables were non-normally distributed. The skewness can easily be determined via the large ratio between the standard deviation and the mean (see Table 1). The 1335 gamblers who reported their subjective gambling expenditure were significantly different with respect to all variables (see Table 1).

**Table 1:** Key variables comparison of survey responders who provided estimates to how much they spent gambling (n = 1335) with the total sample of online gamblers (n = 17,742)

	Total sample (n = 17,742)			Responders (n = 1335)			*p*
	Mean	Median	SD	Mean	Median	SD	
Age (years)	41	39	13	45	45	13	<0.001
% Female	31	0	46	27	0	44	<0.001
GGR (NOK)	1563	701	14,148	2027	1027	6878	<0.001
Amount bet	12,143	1220	37,220	13,554	1941	38,960	<0.001
Customer tenure (months)	94	107	38	107	112	35	<0.001

In addition to being asked about their total gambling expenditure, players were asked whether they thought they had won or lost across all *Norsk Tipping* games played during May 2015 (both online and land-based). Out of the 1335 players, 50 players said that overall they had won (3.7 %). In actuality, 71 players won more money overall in May 2015 (5.3 %). Table 2 reports the comparison between subjective information (columns) and the objective data (rows). The numbers in brackets report the expected frequency given that there is no correlation. Out of the 71 players who actually won more than they lost during May 2015, only 18 of them (subjectively) said they actually won more than they lost. However, overall, there was an association between the subjective and objective data (*X* ^*2*^ = 97, *df* = 1, *p* < 0.001). (i.e., players were aware to some extent of their overall outcome of whether they won or lost money overall). This corresponds to an effect size measured via Cramer’s V of 0.27. Values between 0.1 and 0.3 are regarded as small.

**Table 2:** Comparison between subjective and objective gambling expenditure related to overall win or loss during a one-month period by players (n = 1335)

	Subjective		
	Won	Lost	Total
*Objective*			
Won	18 (3)	53 (68)	71
Lost	32 (47)	1210 (1195)	1242
No Activity	0 (1)	22 (21)	22
Total	50	1285	1335

Players were also asked to estimate specifically how much money they had won or lost overall during May 2015. Table 3 shows the differences between the objective and subjective data. In all subsequent analysis, negative estimated values indicate a win and positive estimated values indicate a loss from the players’ perspective. It is clearly visible that some players reported extremely high values. The biggest estimated win was reported to be 10,000,000 NOK and the largest estimated loss was reported to be 1000,000,000 NOK.

**Table 3:** Comparison between subjective and objective gambling expenditure (NOK) by players (n = 1335)

	Estimated value	Actual value (GGR)	Estimated value (cleaned)	Bias	Bias/average. GGR
Min.	−10,000,000	−122,737	−123,000	−123,400	−200.00
Q1	400	448	400	−725	−0.58
Median	1000	1027	1000	−105	−0.15
Mean	761,900	2027	2952	925	0.43
Q3	2000	2461	2000	400	0.39
Max	1,000,000,000	159,845	160,000	159,100	209.20

These extreme values are highly unlikely to be true and can obviously be regarded as outliers. However, the question remains concerning how to deal with these extreme values. Consequently, it was decided to replace all subjective values that were larger than the largest actual value, and all subjective values that were smaller than the smallest actual value with the minimum actual (−123,000 NOK) and maximum actual value (160,000 NOK) respectively. The actual value (GGR) is the amount lost or won that players were supposed to estimate. In reality, no player won more than 123,000 NOK or lost more than 160,000 NOK in the month of May across all game-types. The present authors thought that these were reasonable values to impute for the most extreme subjective values. The third column (Table 3) reports the distribution of the estimated value after data cleaning. Only the cleaned self-reported gambling expenditure was used for further analysis. Non-parametric statistics were used due to the highly skewed distributions. The median estimated gambling expenditure amounts to 1000 NOK and is similar to the median actual expenditure that amounts to 1027 NOK. Also the 25 % percentile (Q1) and the 75 % percentile (Q3) are also similar. This means, that on average, players reported losing about 1000 NOK in the month of May 2015 whereas in reality they lost a bit more (1027 NOK). However, one has to be careful when interpreting averages across the player base and not on an individual player-level.

The bias reports the deviation between the estimated value and the actual value (estimated value minus the actual value). In case of a reported as well as an actual loss, a positive bias corresponds to an overestimation of the amount lost. In case of a reported and actual win, a positive bias corresponds to an underestimation of the amount won. A negative bias indicates the underestimation of the loss in the case of a reported loss as well as an actual loss. A negative bias can also indicate the overestimation of the win in case of a reported win as well as an actual win. The median bias was −105 NOK and as most players actually lost, this means that they underestimated their losses. Therefore, half of the players underestimated their losses by at least by 105 NOK. It is difficult to determine what magnitude of the bias is significant. A bias of 10 NOK might mean something different for a player who spends 100 NOK monthly compared to a player who spends 1000 NOK monthly. For that reason, another metric was calculated where the bias was divided by the average GGR from the previous three months gambling. This resulting metric reports the bias in average spending units and is also reported in Table 2. The median value of −0.15 means that on average, players underestimate their losses by 15 % of their average loss. Again, the mean values cannot be interpreted due to the skewed nature of the distributions.

The most natural way of determining whether the metrics are related is the correlation coefficient. The linear (Pearson) correlation between the actual expenditure and the self-reported expenditure was 0.35 that corresponds to an explained variance of 12 %. The Spearman correlation that uses ranks (rather than the actual values) was 0.61. Correlation quantifies the degree to which two variables are related. But a high correlation does not automatically imply that there is good agreement between two variables. The correlation coefficient can be misleading when assessing agreement, because it evaluates only the linear association of two sets of observations (Giavarina 2015). The Pearson correlation assesses the strength of a relation between two variables, not the agreement between them. Similarly, r-square only tells us the proportion of variance that the two variables have in common. Altman and Bland (1983) introduced a plot to more accurately describe the agreement between two variables. This plot helps to identify patterns and whether there is a systematic bias in the agreement of two variables. The resulting graph is a scatterplot in which the difference between two variables on the Y-axis is plotted against the average of the two variables on the X-axis.

**Fig. 1:**
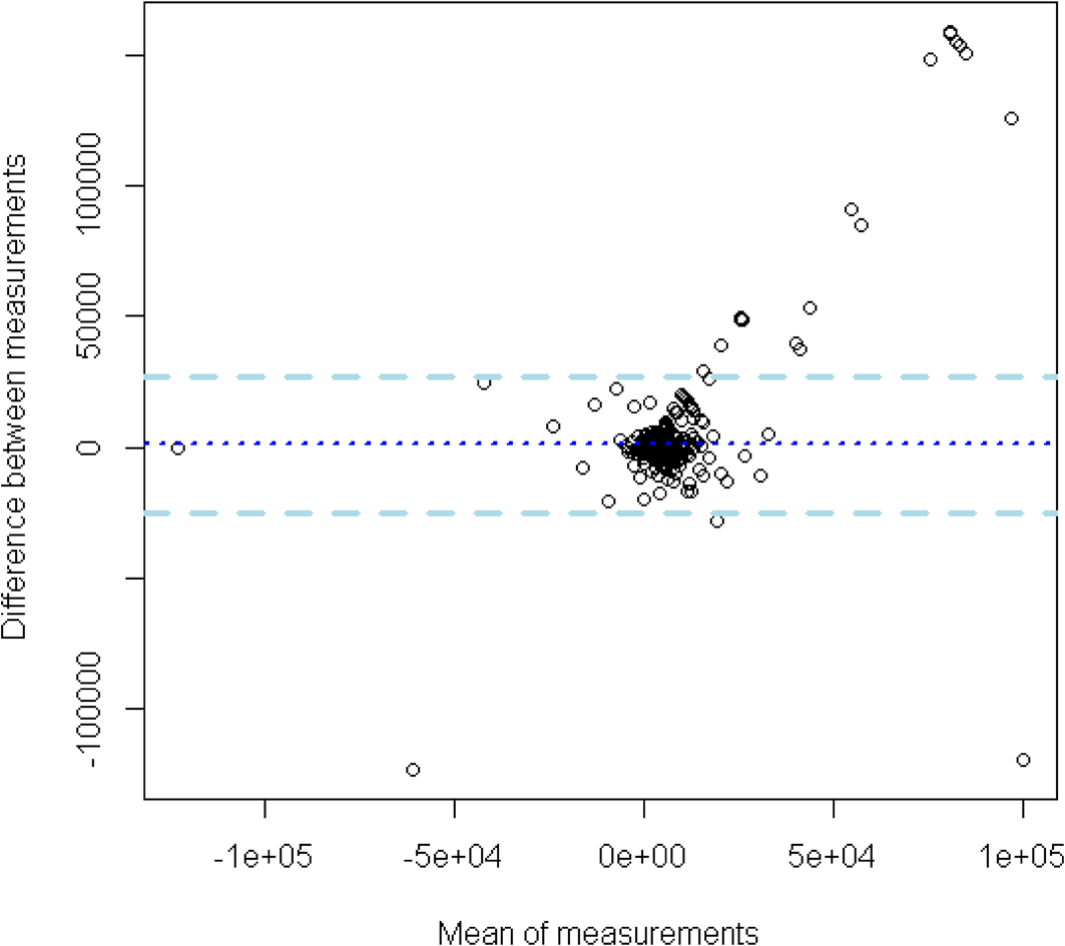
Bland Altman plot of the subjective data and objective data of players (n = 1335)

**Fig. 2:**
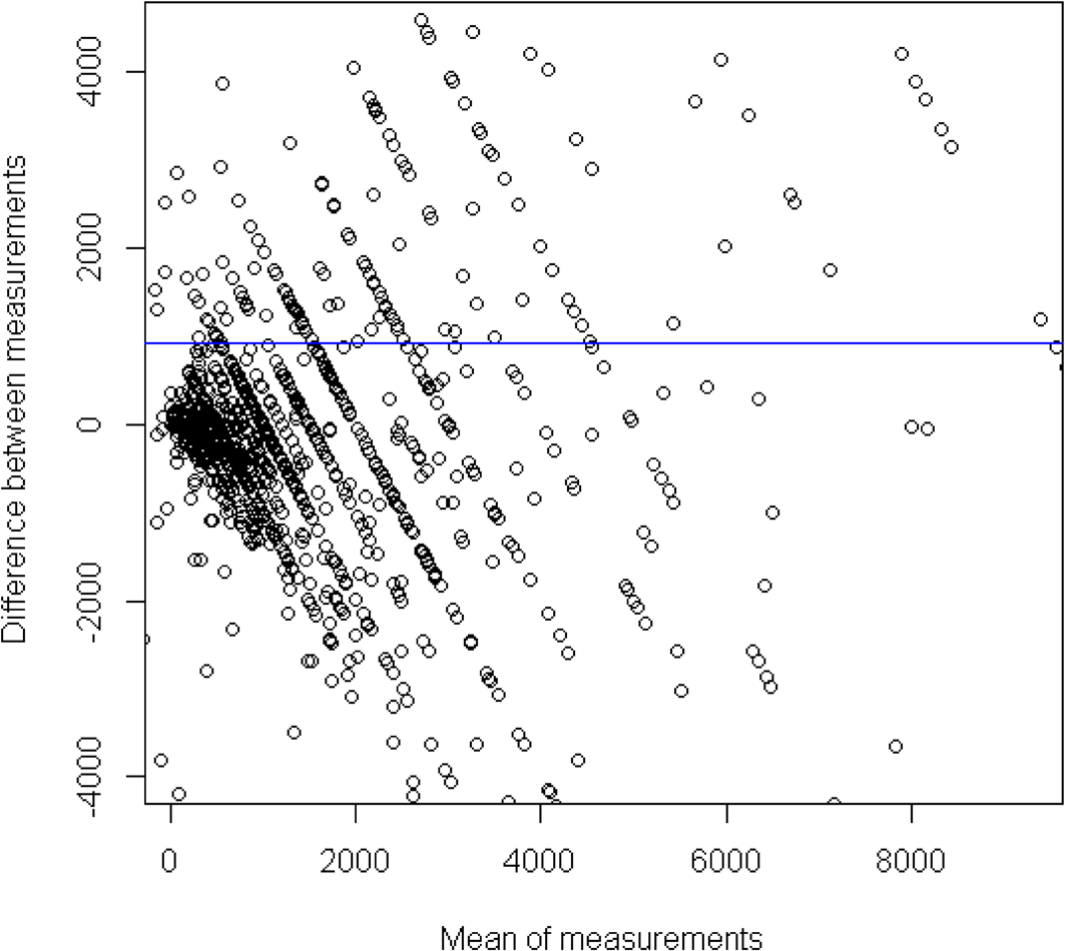
Bland Altman plot of the subjective data and objective data of players (n = 1335) with a limited range of the x-axis metric and the y-axis metric

The Bland–Altman plot of the subjective data and the objective data can be seen in Fig. 1. Each of the dots represents one of the 1335 players where the difference between the two metrics is on the Y-axis and the mean for the two values is on the X-axis. If there is no systematic estimation bias all the dots would scatter randomly across the average bias which is depicted by the dotted line (925). It is difficult to determine whether there is a systematic estimation bias as the metric is highly skewed and most dots are compressed in a narrow range. What can be determined is that the bias is very high in the area of large values. Most of the values in the right half of Fig. 2 are outside of the confidence interval depicted by the dashed lines. This suggests that players with higher losses tend to have more difficulty estimating their gambling expenditure.

For that reason, it is helpful to restrict the range of the values. Figure 2 reduces the range of the Y-axis and the X-axis at the 5th and 95th percentile. This means that the 10 % most extreme players are omitted from the analysis. It is clearly visible that the variance of the bias (Y-axis) increases as the mean between the subjective and objective metric increases. As already assumed from Fig. 1, the estimation bias increases with the intensity of gambling. Players who spend more money gambling also appear to have more trouble estimating their gambling expenditure accurately.

Another important question is whether the estimation bias is associated with other variables such as the game type preference, age, and/or gender. Table 4 reports the bias in GGR units for groups of players with different game type preferences. Therefore, the bias between the subjective and objective money spent information has been being divided by the average GGR over the past 3 months. This can be regarded as a form of normalization. A player’s corresponding value thus reports the bias as a percentage of this player’s average expense. The median value of the bias in average GGR units is −0.15 and was reported in Table 3. Consequently, on average, players are underestimating their losses by 15 % of their average GGR. Table 4 reports that casino players underestimate their losses by 27 % of the average GGR and was statistically significant. All other types of players do not deviate significantly from the average player.

**Table 4:** Distribution of the normalized bias (bias divided by average GGR) for each game type preferences category among players (n = 1335)

	Median	*p* value (%)
Lottery	−0.16	63
Casino	−0.27	2
Scratchcards	−0.01	8
Sports betting	−0.20	61
VLTs	−0.16	90

Braverman et al. (2014) suggested classifying an estimation as correct if the bias between the subjective and objective value was in the range of the average GGR. This means that the aforementioned metric bias/average (GGR) is classified as correct if it is between −1 and +1. If it is smaller than 1 it is classified as unfavorable (underestimation of losses or overestimation of wins), and if it is larger than 1 it is classified as favorable (overestimation of losses or underestimation of wins). Only 70 out of 1242 players actually won in reality. If a player loses 100 NOK and estimates the loss to be 80 NOK and has lost 110 NOK on average over the last 3 months, than the estimate can be regarded as correct because the bias/average (GGR) (−20/110) is within the range of −1 and +1. Table 5 reports the percentage of correct, unfavorable, and favorable estimates across the different game preferences.

**Table 5:** Bias classification across game-preferences among players (n = 1335)

	Unfavorable (%)	Correct (%)	Favorable (%)	*p*
Lottery	7	81	12	0.001
Casino	13	71	16	0.045
Scratchcards	10	64	26	0.009
Sports betting	16	63	21	0.03
VLTs	11	74	15	0.35
Total	9	74	17	

The last row reports the percentages across all players. In total, 74 % players estimated their expense within the defined range (average GGR). A total of 9 % of the players underestimated their losses or overestimated their wins, and 17 % of the players overestimated their losses or underestimated their wins. Lottery players have the highest percentage of correct estimates, whereas scratchcard players and sports bettors have the lowest. Scratchcard players have the highest percentage of favorable bias and sports bettors have the highest percentage of unfavorable bias. Only the distribution among video lottery terminal (VLT) players is not significant.

Tables 4 and 5 report different metrics. Whereas Table 4 reports the bias in average GGR units, Table 5 reports the categorized value. Table 4 reports the ratio of the bias (objective vs. subjective loss) and the average GGR for each game-type preference. This is a normalized bias that is independent of the level of play expressed in average GGR units. Whereas all types of gambler underestimated their losses, only casino players significantly deviated from the overall distribution. Table 5 categorizes the normalized bias for each player. If the bias is within the average GGR, then a player’s estimation is categorized as “correct”, otherwise it is either an “underestimation” or “overestimation”. Apart from VLT players, all types of players deviated significantly from the overall distribution. Casino players and sports bettors showed the largest percentage of unfavorable estimation (i.e., they underestimated the amount of money they had actually spent gambling).

The survey also included the question “*How aware are you of your own gambling?”* and a total of 458 gamblers responded (131 answered “yes”, 302 answered “no” and 25 answered “I don’t know”). Due to the non-normal distribution of the bias, a Kruskal–Wallis test was performed. The estimation bias was not significantly different across the three categories (*X* ^*2*^ = 0.088, *df* = 2, *p* = 0.96).

Finally, a multiple regression with loss, game type preference, gender, and age as independent variables, and bias as the dependent variable was performed. The amount lost as well as the amount of money gambled in sports betting and VLTs was significant and 12 % of the variance was explained. This is in line with the results from the Bland–Altman analysis that also showed that estimation bias increases as a function of increased losses. However, gender and age were not significant. A multiple regression with the estimation bias in average GGR units only yielded an explained variance of 0.3 % and the only significant variable was the amount of money lost on VLTs. This means that the estimation bias is almost exclusively a function of the money spent. It appears that there is little correlation with any other type of behavior, apart from monetary gambling intensity.

## Discussion

The present study is one of only two studies that have directly compared objective versus subjective data in relation to gambling spend (the other being Braverman et al. 2014). The main findings were that the estimated loss self-reported by gamblers was correlated with the actual objective loss. The results also showed that players (to some extent) appeared to be able to estimate whether they had won or lost overall during the month’s playing period. In addition, the estimation bias increased with the intensity of play and the types of games played are predictive of the magnitude of the estimation bias. Put more simply, these findings suggest that players with higher losses tend to have more difficulty estimating their gambling expenditure (i.e., players who spend more money gambling also appear to have more trouble estimating their expenses accurately). This finding was also reported in the study by Braverman et al. (2014). The hypothesis that players who engaged in high event frequency games (e.g., casino-type games) would be less accurate in estimating their losses compared to those players who engaged in low event frequency games (e.g., lottery games) was confirmed. Players’ subjective awareness of their own gambling did not seem to be associated with the ability to estimate their personal gambling expenditure. The present study also found that those providing subjective estimates of their gambling expenditure played more intensely, were older, and less likely to be female compared to those who did not.

The correlation between the subjective and objective information was 0.35 and means that players who reported large losses also tended to lose more, and players who reported small losses also lost less. The Bland and Altman analysis reported that players who lost more in actuality also had more trouble estimating their monetary losses accurately. The Bland–Altman analysis does not contradict this and simply examines the data in a different way. These two findings are not contradictory. They are just the consequence from the application of two different statistical methods and both hold true. However, the latter insight cannot be made using a correlation analysis.

In the study by Braverman et al. (2014), *bwin* online players estimated their 3-month losses and 12-month losses, respectively. They concluded that players were more accurate when asked about their short-term losses (3 months) compared to long-term losses (12 months). In the present study, players were asked about their most recent (1-month) losses or wins. Based on Braverman et al.’s (2014) findings, the percentage of correct estimates should be much larger in the present study. This is indeed the case because Braverman et al. (2014) reported that 49 % of players who estimated their 3-month losses were unfavorably biased which is in stark contrast to the numbers in the present study, which are much lower. They also reported that 23–48 % of players overestimated their wins or underestimated their losses, which is also much higher than the numbers reported in the present study.

The correlation between the actual expenditure and the self-reported expenditure was 0.35 (explaining 12 % of the variance). Unfortunately, there is no comparative value from any gambling research study. However, Hagstromer et al. (2010) reported that the correlations between self-reported physical activity and objective measurements range from 0.07 to 0.36. The present study’s correlation coefficient of 0.35 is thus in the upper range, meaning that players are at least as good in estimating their expenditure as people are estimating their physical activity. The Spearman correlation that uses ranks (rather than the actual values) was 0.61. The fact that the Spearman correlation is larger than the Pearson correlation demonstrates that the correlation between the self-reported expenditure and the objective expenditure is not linear in nature.

Players systematically underestimate their losses or overestimate their wins. The degree of bias between the estimate and the actual value is mostly a function of monetary gambling intensity. Analysis also showed that the game preference is correlated with the degree of bias. However, due to the nature of the games, sports betting, playing lottery games, and casino gambling are quite different with respect to the average expenditure. Variables such as age and gender do not appear to be associated with estimation bias. Players who claim to be aware of their own gambling do not estimate their expenditure more accurately than players who claim not to be aware of their own gambling. These results raise the question how studies that make assumptions about playing behavior based on self-recollected information should be interpreted (e.g., Gainsbury et al. 2014).

The present study is not without its limitations. The data were collected from only one online gambling operator (i.e., *Norsk Tipping*) and from one country (i.e., Norway) and may not be representative of gamblers from other countries or those that gamble with other operators. Furthermore, those that responded to the expenditure questions (only 7.5 % of the total sample surveyed) were not representative of the total sample. More specifically, the present study found that there were differences between those that provided subjective data on their gambling expenditure compared to the objective data of the whole sample. However, the fact that those who provided subjective estimates of their gambling expenditure played more intensely, were older, and less likely to be female is likely to be due to the fact that players who frequent the site more often are more likely to engage with any kind of feature on the gambling website. Put simply, more intense players were more likely to notice the request to participate in the study. Additionally, players were not aware of the survey questions prior to viewing them. This means that players did not decide to not participate because of the nature of the survey questions. In sum, the findings demonstrate that caution is warranted when using self-report data relating to amount of money spent gambling in studies that only use self-report data (even in large-scale nationally representative epidemiological studies).
